# Potential Benefits of Omega-3 Fatty Acids in Non-Melanoma Skin Cancer

**DOI:** 10.3390/jcm5020023

**Published:** 2016-02-04

**Authors:** Homer S. Black, Lesley E. Rhodes

**Affiliations:** 1 Department of Dermatology, Baylor College of Medicine, Houston, TX 77030, USA; 2 Photobiology Unit, Dermatology Centre, University of Manchester, Salford Royal Hospital, Manchester M6 8HD, UK; Lesley.E.Rhodes@manchester.ac.uk

**Keywords:** skin cancer, omega-3 fatty acids, ultraviolet radiation, prostaglandins, immune modulation

## Abstract

Considerable circumstantial evidence has accrued from both experimental animal and human clinical studies that support a role for omega-3 fatty acids (FA) in the prevention of non-melanoma skin cancer (NMSC). Direct evidence from animal studies has shown that omega-3 FA inhibit ultraviolet radiation (UVR) induced carcinogenic expression. In contrast, increasing levels of dietary omega-6 FA increase UVR carcinogenic expression, with respect to a shorter tumor latent period and increased tumor multiplicity. Both omega-6 and omega-3 FA are essential FA, necessary for normal growth and maintenance of health and although these two classes of FA exhibit only minor structural differences, these differences cause them to act significantly differently in the body. Omega-6 and omega-3 FA, metabolized through the lipoxygenase (LOX) and cyclooxygenase (COX) pathways, lead to differential metabolites that are influential in inflammatory and immune responses involved in carcinogenesis. Clinical studies have shown that omega-3 FA ingestion protects against UVR-induced genotoxicity, raises the UVR-mediated erythema threshold, reduces the level of pro-inflammatory and immunosuppressive prostaglandin E2 (PGE_2_) in UVR-irradiated human skin, and appears to protect human skin from UVR-induced immune-suppression. Thus, there is considerable evidence that omega-3 FA supplementation might be beneficial in reducing the occurrence of NMSC, especially in those individuals who are at highest risk.

## 1. Introduction

Considerable interest has been focused on the potential health benefits of omega-3 fatty acids (FA) on a range of human diseases. This interest arose from a series of reports in which high dietary intake of these unsaturated FA among Greenlandic West Coast Eskimos was specifically associated with low incidence of ischemic heart disease, and inflammatory symptoms, in general [1,2,3]. Whereas the major focus has been on cardiovascular disease [4,5,6], studies have been extended to type II diabetes and the metabolic syndrome, inflammatory bowel disease, rheumatoid arthritis, renal disease, systemic lupus erythematosus, and osteoporosis [7,8].

Dietary lipids have also been implicated in the development of several kinds of cancer, e.g., breast, lung, bowel, bladder, pancreatic, and prostate [9,10,11,12]. Whereas, omega-3 FA have generally shown positive effects on cardiovascular disease, studies are equivocal for human cancers [13,14]. Among 43 risk ratios calculated across 19 cohorts for 11 different types of cancer and 5 different ways to assess omega-3 FA consumption, only four were significant, and it was concluded that omega-3 FA do not reduce overall cancer risk [14]. A systematic review involving 20 cohorts and using up to 6 different ways to categorize omega-3 FA consumption similarly arrived at the conclusion that, overall, there was not a significant association between omega-3 FA and cancer incidence and that dietary supplementation was unlikely to prevent cancer [15].

Although women with high intake ratios of marine omega-3 FA, relative to omega-6 FA have been found to have a reduced risk of breast cancer, not all case-control and cohort studies are in agreement [16]. In the first meta-analysis previously referenced [14], five estimates of risk for breast cancer did not show a significant association, and no effects were found for cancers of the aero-digestive tract, bladder, colorectum, ovary, pancreas or stomach, or for lymphoma. While a recent study observed that high levels of serum phospholipid omega-3 FA (a biomarker) were associated with a large *increase* in the risk of high-grade prostate cancer [17], subsequent systematic review and meta-analysis, including 12 studies of self-reported dietary intake of omega-3 FA and 9 biomarker studies, failed to find an association between omega-3 FA and prostate cancer [18]. These ambiguities require clarification and undoubtedly will require randomized, double-blinded intervention trials.

Inflammatory processes are involved in initiation, promotion, and progression stages of cancer and herein rests the rationale upon which omega-3 FA might be expected to reduce cancer risk [19]. In this regard, there has accrued a considerable body of evidence, albeit circumstantial at this point, that omega-3 FA could reduce cancer risk for the most common of cancers, *i.e.*, skin cancer. The American Cancer Society [20] estimates that over 3.5 million cases of skin cancer will occur this year in the United States alone. The evidence to support a beneficial outcome for omega-3 FA supplementation on non-melanoma skin cancer (NMSC) is presented herein.

## 2. Essential Fatty Acids

Linoleic acid (LA) and α-linolenic acid (ALA) cannot be synthesized by humans and are, thus, considered essential and must be supplied in the diet. These essential fatty acids (EFA) are the precursors of the omega-6 and omega-3 series of FA, respectively. These FA are often abbreviated by their chemical designation, e.g., LA is 18:2*n*-6 where 18 indicated the length of the carbon chain, the 2 represents the number of double bonds and the *n*-6 indicates that the first of the double bonds begins at the sixth carbon atom from the methyl end of the carbon chain. ALA is abbreviated as 18:3*n*-3, the *n*-3 signifies that the first double bond is at the third carbon from the methyl end of the chain. Longer chain polyunsaturated FA (PUFA) can be synthesized in humans from their respective precursor EFA (LA or ALA) through a series of elongation (addition of two carbon atoms) and desaturation (addition of a double bond) enzymatic reactions. The two series of EFA cannot be inter-converted in humans and thus compete for these enzymes. Because Western diets may contain 15–20 times more LA than ALA, greater levels of long- chain omega-6 FA (Arachidonic acid, 20:4*n*-6) result. Consequently under certain dietary conditions supplementation with EPA (Eicosapentaenoic acid, 20:5*n*-3) and DHA (Docosahexaenoic acid, 22:6*n*-3) may be essential for maintenance of good health.

**Figure 1:**
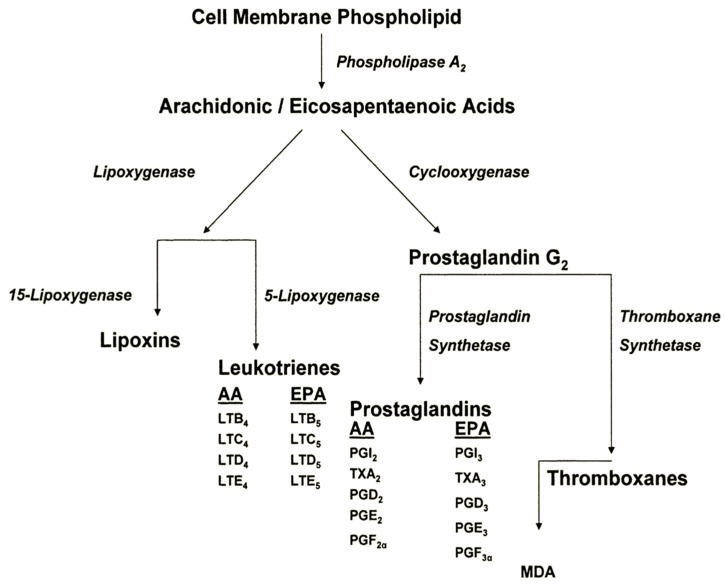
Differential eicosanoid metabolism from omega-6 and omega-3 FA sources. Arachidonic acid, 20:4*n*-6 (AA), is metabolized via lipoxygenase and cyclooxygenase pathways. Eicosapentaenoic acid, 20:5*n*-3 (EPA) acts as a competitive inhibitor to the cyclooxygenase enzyme complex with AA and produces different leukotriene and prostaglandin oxidation products. Malondialdehyde (MDA) is a product of prostaglandin and thromboxane metabolism and is commonly used as a measure of lipid peroxidation.

Not only do these two series of EFA compete for elongase and desaturase enzymes, but they also compete with the cyclooxygenase (COX) and lipoxygenase (LOX) enzymes and differentially influence the flux of metabolites through these pathways. These oxidative metabolites differ in hormonal potency. The omega-6 FA derived products are more active than their omega-3 FA counterparts. Some of these metabolites are known to influence tumor biology. PGE_2_, derived from omega-6 FA oxidation via the COX pathway, acts as a tumor promoter and has been associated with aggressive tumor growth patterns in both basal cell carcinoma (BCC) and squamous cell carcinoma (SCC) in humans [21]. On the other hand, omega-3 FA compete with omega-6 FA for binding sites on COX and inhibit the production of PGE_2_, resulting in higher levels of the less potent PGE3. Omega-3 FA may also shunt potential PG precursors through the LOX pathway, resulting in products that inhibit tumor growth and in products that are involved in immune surveillance [21,22,23]. A simplified schema of eicosanoid metabolism is illustrated in Figure 1. Thus, the COX and LOX pathways, with their bioactive intermediates, provide a strong rational foundation for the cancer preventive potential of omega-3 FA [24].

## 3. Evidence for Participation of Dietary PUFA in UVR-Induced Skin Cancer

### 3.1. Animal Studies

The first report that dietary fat could potentiate UVR-carcinogenesis came in 1939 [25]. With the advent of World War II, this avenue of research lay fallow for nearly 45 years until this lead was followed-up in a series of studies that demonstrated that an approximate linear relationship occurred between PUFA (dietary corn oil that contained roughly 50% omega-6 FA) and UVR-carcinogenic expression [26,27]. Increasing dietary levels of omega-6 FA shortened the tumor latent period and increased tumor multiplicity. Partial hydrogenation of the PUFA resulted in a marked inhibition of carcinogenesis [26]. Reeve *et al.* [28] found that feeding a totally hydrogenated PUFA completely abolished UVR-carcinogenic expression while those animals fed the normal PUFA exhibited 100% tumor incidence. Furthermore, when the diet of animals fed the hydrogenated fat was reconstituted with a normal mixed fat, large numbers of tumors rapidly appeared. The authors suggested that UVR initiation of tumors had not been prevented by lack of PUFA, but that the EFA deficiency held the appearance of tumors in abeyance, probably at the promotion stage of carcinogenesis. Confirmation that omega-6 FA exerted their influence principally at the post-initiation stage of carcinogenesis came from cross-over feeding studies [29]. Animals were placed on a defined, isocaloric diet containing high (12% *w*/*w*) and low (0.75%, *w*/*w*) levels of corn oil. At completion of a regimen of UVR, and before tumors appeared, some diets were crossed to the contravening diet, e.g., high to low fat and low to high fat. Incidence curves and tumor multiplicity analysis provided confirmation that diets containing high levels of omega-6 FA enhanced UVR-carcinogenic expression and that enhancement occurred during the post-initiation, or promotion/progression, stages of carcinogenesis. Importantly, crossing from a high fat to a low fat diet after a cancer causing dose of UVR had already been administered, negated the exacerbating influence of high fat diets. This finding provided the rationale upon which a low-fat dietary intervention might act to ameliorate cancer expression.

Contrary to the tumor promoting effects of omega-6 FA, animals fed a diet containing menhaden oil as lipid source exhibited a marked *inhibition* of UVR-carcinogenic expression [30]. Menhaden oil is rich in omega-3 FA. Unlike omega-6 FA, cross-over feeding studies indicated that omega-3 FA exert their principal anti-cancer effects during the initiation stage of carcinogenesis. Animals fed with the omega-3 FA diet throughout the study exhibited an increased tumor latent period and decreased tumor multiplicity compared to animals receiving an equivalent level of corn oil (rich in omega-6 FA).

As noted previously, omega-3 FA compete with omega-6 FA for active sites on COX, a major enzyme in the eicosanoid cascade [24,31]. As such, the level of pro-inflammatory and immune modulating omega-6 FA metabolites is reduced. As dietary omega-6 FA increase, the plasma PGE_2_ level increases. Omega-3 FA intake reduces PGE_2_ levels approximately 7-fold in comparison to an equivalent level of omega-6 FA [32]. These data support the thesis that omega-6 and omega-3 PUFA differentially influence not only PGE_2_ levels, but other pro-inflammatory and immune-modulating intermediates of the COX and LOX pathways.

Supporting evidence for a role of omega-3 FA in carcinogenesis has recently been acquired from studies with a transgenic mouse model designated *fat-1* [33]. The *fat-1* transgenic mice are capable of producing omega-3 FA from omega-6 FA, *i.e.*, the transgenic has received a gene encoding an omega-3 FA desaturase that converts omega-6 FA to omega-3 FA. This results in abundant omega-3 FA and reduced omega-6 FA in the animals’ tissues without the need for omega-3 FA supplementation and eliminates many of the confounding variables encountered in dietary studies. With regard to skin tumorigenesis, Xia *et al.* [34] showed that there was a dramatic reduction of melanoma formation and growth when *fat-1* mice were injected with B16 melanoma cells, compared to their non-transgenic littermates. The levels of omega-3 FA and metabolite PGE_3_ were much higher in the transgenic animals and the omega-6/omega-3 FA ratio much lower. This transgenic model should be invaluable in future studies to elucidate the role and mechanism(s) of effects of omega-3 FA in NMSC.

As alluded to earlier, previous studies had indicated that carcinogenesis might be modulated immunologically and that this influence might occur at the promotion stage [32]. Notably, the systemic alteration induced by UVR that suppresses an animal’s ability to reject highly antigenic UVR-induced allergens occurs during the chemical promotion stage of carcinogenesis [35]. Reeve *et al.* [28] had already shown that feeding mice an EFA (omega-6 FA) deficient diet inhibited the appearance of UVR-induced tumors and suggested that this inhibition might be due to the lack of eicosanoid precursors that, in turn, might prevent UVR induction of the immune state. In the case of omega-6 FA, this would be deficient levels of PGE_2_, the gate-keeper for other eicosanoids. This would account for protection observed from UVR-initiated tumor outgrowth. Chung *et al.* [36] had shown that T-cell function was PGE_2_ dependent and that UVR-induced suppression of contact hypersensitivity (CHS) was abrogated by treatment with an inhibitor of PG synthesis.

It was subsequently shown that plasma PGE_2_ levels were directly related to omega-6 FA dietary intake, *i.e.*, the highest level of PGE_2_ occurring with the highest level of omega-6 FA intake [32]. This, in turn, induced the greatest exacerbation of UVR carcinogenic expression. Importantly, omega-3 FA provided striking protection against UVR-induced immunosuppression. This observation was subsequently confirmed [37]. Delayed type hypersensitivity (DTH) and CHS are both regulated by T-cell function and share common pathways with immunological tumor rejection. DTH in UVR-irradiated animals is dramatically suppressed in animals fed high levels of omega-6 FA when compared to those receiving low levels of the FA or those receiving omega-3 FA [32,38].The ability of an animal to reject a transplanted tumor was related to the level of omega-6 FA intake. Moreover, the tumor rejection time for animals fed high levels of omega-6 FA was three times longer than animals fed low levels of the omega-6 FA and occurred at a time when high omega-6 FA had been shown to exacerbate primary tumor expression [38].These studies suggest that one potential mechanism of omega-3 FA inhibition of carcinogenic expression is via immune modulation [31].

In summary, the following sequence of observations support the thesis that omega-6, -3 PUFA metabolism, through the LOX and COX pathways, leads to differential metabolites that influence inflammatory and immune responses involved in UVR-carcinogenesis:

1. Increasing levels of dietary omega-6 FA *exacerbate* UVR carcinogenic expression, with respect to both shortened tumor latent period and increased tumor multiplicity.
2. Dietary omega-3 FA *inhibit* UVR carcinogenic expression.
3. Omega-6 FA exert their principal effect upon the post-initiation, or promotion/progression stages of UVR carcinogenesis.
4. Omega-3 FA appear to exert their principal effects during the initiation stage of the carcinogenic continuum.
5. Pro-inflammatory and immunosuppressive PGE_2_ levels are increased linearly as dietary omega-6 FA levels increase.
6. Pro-inflammatory and immunosuppressive PGE_2_ levels are dramatically reduced by dietary omega-3 FA intake.
7. Dietary omega-6 FA suppress the immunologic responses involved in tumor transplant rejection and the immunologic pathways involved in DTH and CHS.
8. Dietary omega-3 FA inhibit UVR-induced suppression of DTH and CHS.

### 3.2. Clinical Studies

The experimental studies employing a high-fat diet to low-fat diet cross-over, even after a cancer causing dose of UVR had been administered, negated the exacerbating influence of the high-fat diet and provided a rationale for undertaking a clinical intervention trial. This trial, involving 133 skin cancer patients, of whom 115 completed the two year study, clearly demonstrated that a low-fat intervention reduced the occurrence of NMSC [39,40]. The cumulative rate of occurrence of NMSC (cumulative NMSC/patient/time period) was 0.21 and 0.19 during the first 8-month period of the study and 0.26 and 0.02 (*p* ≤ 0.02) during the last 8-month period for control and intervention arms, respectively. The dietary parameters involved only a reduction in the calories consumed as fat, while maintaining total calorie intake and body weight. Efforts were made to maintain the P/S ratio (polyunsaturated/saturated fat ratio) of patients’ diets going into the trial and there were no increases in omega-3 FA intake. Thus, the influence of fat on MNSC occurrence was primarily that resulting from lowering fat intake, primarily omega-6 FA. Furthermore, the influence of this low-fat intervention was observed early in the study as a significant difference in the number of actinic keratoses (pre-malignant lesions) between control and low-fat diets occurred [41]. Patients in the control arm of the study (no dietary modifications introduced) were found to be at 4.7 times greater risk of having one or more actinic keratoses during the two-year study period than patients in the low-fat intervention arm.

Whereas lower intake of omega-6 FA reduces the risk of NMSC occurrence in skin cancer patients, a population based case-control study showed a consistent tendency toward a lower risk of SCC with higher intakes of omega-3 FA [42]. Their data also suggested a tendency for a lower risk of SCC with diets containing high omega-3/omega-6 FA ratios. Although this study was suggestive that omega-3 FA could influence NMSC risk, a number of human studies have provided a physiological rationale to support such a hypothesis. Encouraged by the experimental animal results, a short term supplementation study of mixed omega-3 FA was conducted in humans [43]. The patients received oral capsules of either 4 g/day of mixed omega-3 FA (2.8 g EPA + 1.2 g DHA) or a gelatin placebo. After four weeks there was a statistically significant increase in the minimal erythema dose (MED) to UVB in the active group. Serum triglyceride levels decreased by 40 mg/dL. A second study examined the effects of omega-3 FA supplementation on UVB-induced erythema and lipid peroxidation [44]. This study employed a supplement of 3 g/day of mixed omega-3 FA (1.8 g EPA + 1.2 g DHA) administered over a 3–6 month period. The MED rose progressively with increasing time of omega-3 FA supplementation, and had more than doubled at six months. This increase in MED was accompanied by an increase in epidermal omega-3 FA composition and increased susceptibility to lipid peroxidation. The MED had returned to baseline two and a half months after omega-3 FA supplementation was halted.

As noted earlier, a number of cytokines and PG have been shown to be modulated by omega-3 FA. When human keratinocytes were cultured in the presence of omega-3 FA, TNF-α and IL-1α secretion was induced and PGE_2_ and IL-6 level reduced [45]. Subsequently, further *in vitro* keratinocyte studies showed that EPA and DHA each inhibited basal and UVR-induced IL-8, a chemokine pivotal to UVR- induced skin inflammation and which exhibits pro-carcinogenic activity [46]. However, a double- blind, randomized trial of 28 patients supplemented with 4 g/day of 95% of ethyl esters of EPA or oleic acid for three months found no evidence that the MED response evoked by omega-3 FA was mediated by the pro-inflammatory cytokines IL-8, TNF-α, IL-6 or IL-1β. In contrast, there was a notable and significant reduction in cutaneous PGE_2_, the pro-inflammatory and immune-suppressor mediator [47]. Further, lipidomic analysis was performed in a human intervention trial of EPA-rich omega-3 FA, quantifying impact of supplement on eicosanoid levels in skin blister fluid [48]. This showed a significant reduction in the ratio of PGE_2_: PGE_3_ in UVR-exposed skin, accompanied by a reduction in the ratio of the pro-inflammatory and tumor promoting 12-LOX product 12-hydroxyeicosatetraenoic acid (12-HETE): 12-hydroxyeicosapentaenoic acid (12-HEPE), EPA-derived homologue of 12-HETE [48].

A double-blind, randomized intervention examined the impact of oral omega-3 FA on UVR suppression of cell-mediated immunity, assessed through the nickel CHS response [49]. Seventy-nine nickel-sensitive adult females consumed encapsulated omega-3 FA (3.5 g EPA + 1.5 g DHA) or control lipid daily for 3 months, with compliance and skin bioavailability of omega-3 FA assessed by blood [49] and skin [48] assay, respectively. This indicated apparent abrogation of the photo-immunosuppression induced by low level solar simulated ultraviolet radiation (SSR; 95% UVA, 5% UVB) exposure. Following SSR exposure equivalent to ~15 min of midday summer sunlight in Manchester, UK (latitude 53.5° N), on 3 consecutive days, the UVR-suppression of the CHS response was 50% lower in the subjects taking omega-3 FA compared to those taking control.

Previous discussion provides clear evidence that omega-3 FA protect against the clinical sunburn response. Yet, there was no evidence of an EPA effect on direct UVR-induced DNA damage to DNA, *i.e.*, cyclobutane pyrimidine dimer formation in skin [50]. There was, however, protection against UVR induction of cutaneous p53, considered to be a biomarker of DNA damage and which acts as a tumor suppressor gene. In addition, in *ex vivo* UVR-treated peripheral blood lymphocytes, omega-3 FA protected against DNA single-strand breaks [50].

Thus, human studies (and cell culture studies employing human cells) have shown:

1. Omega-3 FA supplementation significantly increases the erythema threshold to UVR.
2. Omega-3 FA modulate a number of cytokines (in human cells *in vitro* only) and eicosanoids that mediate inflammatory and immune responses.
3. Omega-3 FA inhibit certain genotoxic markers of UVR-induced DNA damage, e.g., UVR- induced cutaneous p53.
4. Omega-3 FA abrogate UVR-induced immunosuppression of cell mediated immunity assessed as nickel CHS

## 4. Conclusions

*In Toto*, the results of experimental studies, and the influence of omega-3 FA on UVR-induced erythema, early genotoxic markers and immune-suppression in human trials, suggest that supplementation of these photoprotective nutrients [51] could result, in the longer term, in a reduction in NMSC in humans. Based upon age-adjusted cancer incidence/UVR exposure plots, an omega-3 FA enhanced sun protection factor (SPF), even of the low reported magnitude could reduce incidence of NMSC by as much as 30% [50,51,52]. Neither have observational studies, case-control or prospective cohort studies, provided clear evidence that dietary omega-6 FA or omega-3 FA reduces the risk of NMSC. A recent meta-analysis has been suggestive, but lacked adequate data due to scarcity of trials in this area, to support the hypothesis that omega-3 FA protect against NMSC [53]. For the most part, both case-control and prospective cohort studies have failed to find a relationship between skin cancer incidence with dietary fat intake. Indeed these types of studies are fraught with methodological difficulties resulting from: (1) the complexity of the human diet in a free living population; (2) the difficulties in measuring food intake and analyzing dietary information; (3) the epidemiologist requires assess of dietary patterns that are stable over long periods, *i.e.*, usually years if cancer induction is under study [54].

The authors have previously proposed that the most direct evidence for the preventive potential of omega-3 FA would be achieved through intervention trials in populations with high, and known, risk for NMSC—much in the manner that reduction in the percentage of calories consumed as fat was shown to influence NMSC occurrence in skin cancer patients [24,39,40,41,55]. Caveats to the design of such a study will include consideration of baseline omega-3 FA nutrition at study inclusion [56] and careful monitoring of the diets of study patients to assure that any potential benefits of omega-3 FA supplementation is not diminished by increasing omega-6 FA intake. The relative omega-6/omega-3 FA ratios will determine response and could be monitored by an easily determined parameter such as red blood cell membrane omega-6/omega-3 FA ratios [48,56]. This parameter could also be used to determine adherence to the supplement protocol. It is also important that an adequate level of omega-3 FA supplementation be employed. Omega-3 FA have a high safety profile and a daily intake of *circa* 4 g/day as employed in previous photoprotection studies is envisaged to be adequate. Because of the promising evidence from animal and clinical studies, it is imperative that the potential of omega-3 FA as a preventive agent for NMSC be fully explored.
